# Periprostatic adipose tissue (PPAT) supernatant from obese mice releases anticontractile substances and increases human prostate epithelial cell proliferation: the role of nitric oxide and adenosine

**DOI:** 10.3389/fphar.2023.1145860

**Published:** 2023-07-10

**Authors:** Gabriela Reolon Passos, Mariana G. de Oliveira, Ana Carolina Ghezzi, Glaucia C. Mello, Carlos Arturo Levi D’Ancona, Simone Aparecida Teixeira, Marcelo Nicolas Muscará, Carla Beatriz Grespan Bottoli, Lucilia Vilela de Melo, Eliezer de Oliveira, Edson Antunes, Fabiola Zakia Mónica

**Affiliations:** ^1^ Section of Pharmacology, Department of Translational Medicine, Faculty of Medical Sciences, University of Campinas (UNICAMP), Campinas, Brazil; ^2^ Division of Urology, Department of Surgery, Faculty of Medical Sciences, University of Campinas (UNICAMP), Campinas, Brazil; ^3^ Department of Pharmacology, Institute of Biomedical Sciences, University of Sao Paulo (USP), Sao Paulo, Brazil; ^4^ Institute of Chemistry, University of Campinas (UNICAMP), Campinas, Brazil

**Keywords:** adenosine, benign prostatic hyperplasia, cell proliferation, nitric oxide, obesity, PPAT, prostate

## Abstract

**Background:** The prostate gland is surrounded by periprostatic adipose tissue (PPAT) that can release mediators that interfere in prostate function. In this study, we examined the effect of periprostatic adipose tissue supernatant obtained from obese mice on prostate reactivity *in vitro* and on the viability of human prostatic epithelial cell lines.

**Methods:** Male C57BL/6 mice were fed a standard or high-fat diet after which PPAT was isolated, incubated in Krebs-Henseleit solution for 30 min (without prostate) or 60 min (with prostate), and the supernatant was then collected and screened for biological activity. Total nitrate and nitrite (NOx^−^) and adenosine were quantified, and the supernatant was then collected and screened for biological activity. NOx^−^ and adenosine were quantified. Concentration-response curves to phenylephrine (PE) were obtained in prostatic tissue from lean and obese mice incubated with or without periprostatic adipose tissue. In some experiments, periprostatic adipose tissue was co-incubated with inhibitors of the nitric oxide (NO)-cyclic guanosine monophosphate pathway (L-NAME, 1400W, ODQ), adenylate cyclase (SQ22536) or with adenosine A_2A_ (ZM241385), and A_2B_ (MRS1754) receptor antagonists. PNT1-A (normal) and BPH-1 (hyperplasic) human epithelial cells were cultured and incubated with supernatant from periprostatic adipose tissue for 24, 48, or 72 h in the absence or presence of these inhibitors/antagonists, after which cell viability and proliferation were assessed.

**Results:** The levels of NOx^−^ and adenosine were significantly higher in the periprostatic adipose tissue supernatant (30 min, without prostate) when compared to the vehicle. A trend toward an increase in the levels of NOX was observed after 60 min. PPAT supernatant from obese mice significantly reduced the PE-induced contractions only in prostate from obese mice. The co-incubation of periprostatic adipose tissue with L-NAME, 1400W, ODQ, or ZM241385 attenuated the anticontractile activity of the periprostatic adipose tissue supernatant. Incubation with the supernatant of periprostatic adipose tissue from obese mice significantly increased the viability of PNT1-A cells and attenuated expression of the apoptosis marker protein caspase-3 when compared to cells incubated with periprostatic adipose tissue from lean mice. Hyperplastic cells (BPH-1) incubated with periprostatic adipose tissue from obese mice showed greater proliferation after 24 h, 48 h, and 72 h compared to cells incubated with culture medium alone. BPH-1 cell proliferation in the presence of PPAT supernatant was attenuated by NO-signaling pathway inhibitors and by adenosine receptor antagonists after 72 h.

**Conclusion:** NO and adenosine are involved in the anticontractile and pro-proliferative activities of periprostatic adipose tissue supernatant from obese mice. More studies are needed to determine whether the blockade of NO and/or adenosine derived from periprostatic adipose tissue can improve prostate function.

## 1 Introduction

Anatomically, the human prostate is divided into peripheral, central, and transition zones (McNeal, 1984). Enlargement of the transition zone and an increase in prostate smooth muscle tonus contribute to the progression of benign prostatic hyperplasia (BPH), a non-malignant condition that compresses the prostatic urethra, thereby worsening bladder emptying. BPH is one of the main causes of lower urinary tract symptoms (LUTS) (D’Ancona et al., 2019) and substantially affects the quality of life of elderly men (>65 years old). LUTS is characterized by an increase in micturition frequency, urinary urgency with or without nocturia, incomplete emptying, and postmicturition dribble (Bayliss et al., 1999; Irwin et al., 2008).

The mouse prostate is organized into four distinct lobes (anterior, dorsal, ventral, and lateral) located around the urethra (Oliveira et al., 2016). Although the anatomy of the rodent prostate differs from that of humans, rats and mice are the most frequently used laboratory animals for studying the physiological and signaling pathways in this organ because of their similarities to the human prostatic tissue, in addition to advantages such as the relatively low cost of these models, ease of handling, and the possibility of genetic manipulation (Oliveira et al., 2016; Mónica and Antunes, 2018).

The therapeutic management of LUTS secondary to BPH aims to reduce the contractility of prostatic smooth muscle and/or inhibit prostatic cell growth (Abrams et al., 2013). Co-morbidities such as diabetes, metabolic syndrome, dyslipidemia, and obesity can negatively affect prostatic function, with pre-clinical and clinical studies showing a positive correlation between BPH and obesity (Penson et al., 2011; Calmasini et al., 2017).

Extraprostatic adipose tissue, classified as periprostatic adipose tissue (PPAT), is in direct contact with the human prostate surface. Histological analysis has shown that 57–59%, 44%, and 36% of the right and lateral surfaces, and the anterior region and posterior region of the prostate, respectively, are surrounded by PPAT (Hong et al., 2003). PPAT releases several mediators with a paracrine role (Passos et al., 2021; Rouminguié et al., 2022). For example, PPAT from obese patients increased the proliferation of prostate cancer cells and endothelial cells compared to cells incubated with PPAT obtained from patients who are overweight or lean (Venkatasubramanian et al., 2014). Several studies have also described the presence of intraprostatic adipose tissue (IPAT) (Cohen and Stables, 1998; Herschap et al., 2011). For instance, in 427 patients with prostate adenocarcinoma, the presence of IPAT was observed in 4% of biopsies from the surrounding benign glands and/or small nerves in the peripheral or central zones (Nazeer et al., 2009).

In obese mice, the gene expression of NADPH oxidase 2 (NOX2) and tumor necrosis factor-a(TNF-a) was higher in PPAT from obese mice compared to lean mice, but no functional assays were done to assess whether reactive oxygen species or TNF-a interfered negatively with prostate function (Alexandre et al., 2018). Based on these studies, we hypothesized that PPAT could release substances that interfere with prostate contractility and cell proliferation. In this study, we used functional, biochemical, and molecular assays to examine the effect of PPAT on the reactivity of prostatic tissue from lean and obese mice and on the viability of immortalized human prostatic epithelial cells.

## 2 Material and methods

### 2.1 Reagents

Bovine serum albumin (BSA), 4-(2-[7-amino-2-(2-furyl)[1,2,4]triazolo [2,3-*a*][1,3,5]triazin-5-ylamino]ethyl)phenol (ZM241385, a selective adenosine A_2A_ receptor antagonist), N-(3-(aminomethyl)benzyl)acetamidine (1400W, an inhibitor of inducible NOS), *N*-(4-cyanophenyl)-2-[4-(2,3,6,7-tetrahydro-2,6-dioxo-1,3-dipropyl-1*H*-purin-8-yl)phenoxy]-acetamide (MRS1754, a selective adenosine A_2B_ receptor antagonist), N^w^-nitro-L-arginine methyl ester (L-NAME, a non-selective inhibitor of nitric oxide synthase–NOS), 1H-[1,2,4]oxadiazolo [4,3-a]quinoxalin-1-one (ODQ, an inhibitor of soluble guanylate cyclase), phenylephrine (Phe, the a_1_-adrenoceptor agonist), and 9-(tetrahydro-2-furanyl)-9H-purin-6-amine (SQ22536, an inhibitor of adenylate cyclase) were obtained from Sigma-Aldrich Chemical Co. (St. Louis, MO, United States). Other reagents, including the salts for Krebs-Henseleit solution preparation, were of analytical grade purchased from Merck (Darmstadt, Hesse, Germany) and LabSynth (Diadema, SP, Brazil).

### 2.2 Animals

Male C57BL/6 mice were obtained from the Multidisciplinary Center for Biological Investigation (central animal house) of the State University of Campinas (CEMIB-UNICAMP) and were housed (3/cage) with water and food *ad libitum* in ventilated stands (temperature: 24 ± 1°C; humidity: 55 ± 5%) on a 12 h light-dark cycle (lights on at 6 a.m.) and a wood shaving substrate that was changed twice a week. All animal protocols were approved by the institutional Committee for Ethics in Animal Use (CEUA/UNICAMP protocol no. 4836–1/2018) and followed the *Brazilian Guidelines for The Production, Maintenance, and Use of Animals for Teaching or Research* of the National Council for the Control of Animal Experimentation (CONCEA), as well as the ARRIVE guidelines. When required, the mice were killed with an overdose of isoflurane (>5% in air) followed by cervical dislocation. The total body weight and the weights of the prostate and periprostatic adipose tissue (PPAT) were determined. The tibial length was also determined, and the average length of the left and right tibia was used to normalize the prostate and PPAT weights.

### 2.3 Experimental groups

After reaching 7 weeks of age, the mice were randomly allocated to either a lean group (control) with a regular diet (70% carbohydrate, 20% protein, and 10% fat; Quimtia, Colombo, PR, Brazil) and fed for 12 weeks or an obese group fed with a high-fat diet (HFD; 29% carbohydrate, 16% protein, and 55% fat; PragSoluções, São Paulo, SP, Brazil) for 12 weeks (five to seven animals/group).

### 2.4 Intraperitoneal glucose tolerance test (GTT) and insulin tolerance test (ITT)

Mice were made to fast for 6 h followed by blood sampling from a tail vein for the quantification of the basal glucose concentration using a glucometer (Accu-Chek^®^, São Paulo, SP, Brazil). To analyze GTT, glucose (2 g/kg, intraperitoneal–i. p.) was administered and blood samples were collected 30, 60, and 120 min later for glucose measurements. The area under the glucose concentration-time curve (AUC) was calculated. Thereafter, to analyze ITT, regular insulin (1.5 U/kg, i. p.; Lilly^®^, São Paulo, SP, Brazil) was administered and blood samples were collected every 5 min from 5 to 30 min for glucose quantification. The rate constant for glucose disappearance (ĸITT) was calculated. Both analyses were performed using Excel v.2303.

### 2.5 Histological analysis

After euthanasia, the prostate and surrounding PPAT were removed from lean and obese mice, weighed, fixed in 4% paraformaldehyde, embedded in paraffin, sectioned (5 mm thick sections), and stained with hematoxylin and eosin (HE) for histological examination. Images were acquired with an Axio Imager A2 photomicroscope (Carl Zeiss, Jena, Germany). The area of 10 adipocytes of PPAT per slide was selected and measured using ImageJ Software (Bethesda, Maryland, United States), and the results were expressed as the mean ± SD.

### 2.6 Proliferation assay (Ki-67)

Slides with sections of the prostatic tissue were immersed in 0.3% H_2_O_2_ to block endogenous peroxidases for 10 min at room temperature. Primary rabbit polyclonal anti-Ki-67 (product no. PA5-16785, Invitrogen, Waltham, MA, United States) was diluted (1:1000) in 1% bovine serum albumin (BSA) and applied to the sections overnight at 4°C. The slides were then washed with TBS and incubated with a secondary antibody (goat anti-rabbit antibody, diluted 1:200; Santa Cruz Biotechnology, Texas, United States) for 40 min at room temperature, and the sections were counterstained using HE and photographed with a Zeiss Axio Imager A2 photomicroscope. The images were obtained at ×40 magnification. Cell proliferation was assessed by counting the number of Ki-67-positive nuclei in five microscopic fields for each section, with five non-consecutive sections being examined for each prostate and expressing this number as a percentage of the total number of cell nuclei in these microscopic fields.

### 2.7 Periprostatic adipose tissue (PPAT) and prostate isolation

**FIGURE 1:**
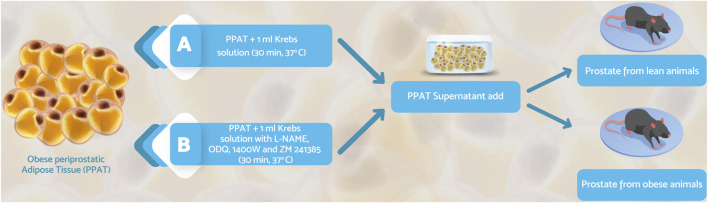
The schematic representation of the bioassay protocols. Periprostatic adipose tissue (PPAT) from obese mice was weighed (∼40 mg), incubated in Krebs-Henseleit solution (30 min, 37°C) with carboxygen, and the supernatant was then collected. Prostate segments from lean or obese mice were incubated (30 min, 37°C without and with PPAT supernatant, in the absence **(A)** or presence **(B)** of NOS inhibitors (L-NAME, a non-selective inhibitor; 1400W, an inhibitor of inducible NOS; ODQ, an inhibitor of soluble guanylate cyclase, and ZM241385, adenosine A_2A_ receptor antagonist).

After euthanasia, the ventral prostate from lean and obese mice was isolated and the PPAT was removed. Two strips of each prostate obtained from lean (strip weight: 1.47 ± 0.38 mg, strip size: 3 ± 0.5 mm n = 6; mean ± SD) and obese (strip weight: 1.36 ± 0.43 mg, strip size: 5 ± 0.4 mm, n = 6, mean ± SD) mice were mounted in a myograph (DNT820MS Muscle Strip System) filled with warm Krebs-Henseleit solution (KHS; composition, in mM: 117 NaCl, 4.7 KCl, 2.5 CaCl_2_, 1.2 MgSO_4_, 1.2 KH_2_PO_4_, 25 NaHCO_3_, and 11 glucose), pH 7.4, and continuously bubbled with a mixture of 95% O_2_/5% CO_2_ at 37°C. A basal tension of 5 mN was applied during a 45 min equilibration period. The isometric force was recorded using a PowerLab 400™ Data Acquisition System (Software Chart, version 6.0, AD Instrument, Milford, MA, United States). Periprostatic adipose tissue obtained from obese mice was weighed (40 mg), filled with the vehicle Krebs-Henseleit solution (KHS), and bubbled with carboxygen (95%O_2_/5% CO_2_) at 37°C for 30 min. After this period, the supernatant was collected and added to isolated prostates. After this period, the supernatant was used for the bioassay protocols (Figure 1, protocol A). Since the amount of PPAT obtained from lean mice was very low and would require many animals to provide 40 mg of PPAT, it was not possible to test PPAT from this group for ethical reasons and because of the cost involved. For this reason, only PPAT supernatant from obese mice was tested in the functional assays. The control tissue refers to strips incubated with 1 ml of KHS alone.

### 2.8 NOx^−^ quantification

#### 2.8.1 Samples preparation

Total nitrite and nitrate (NOx) were quantified in the supernatant in two timepoints: 1) 40 mg of PPAT from obese mice, without prostates, were incubated with KHS, and after 30 min, aliquots were obtained, and 2) prostates from obese mice were isolated, divided into 2 strips, and placed in the chambers. One strip received the PPAT supernatant obtained above, and the other strip received KHS. After 60 min, aliquots of each chamber were collected and stored at -20°C until the NOx quantification. The timepoint of 60 min was chosen because it was the period that the functional assay lasted (30 min of incubation with PPAT or KHS +30 min of the CRC to Phe).

#### 2.8.2 Quantification

For the quantification of nitrite concentration in the PPAT supernatant, the Griess reaction method was used (Herrera et al., 2012). The PPAT supernatant was kept at room temperature with an equal volume of Griess reagent (composed of sulphanilamide plus 0.1% naphthylethylenedihydrochloride in 2.5% phosphoric acid, Sigma Chem Co., St. Louis, MO, United States). The absorbance was measured at 540 nm. The concentration of the PPAT supernatant was calculated using the calibration curve obtained through linear regression between the nitrite concentration and the absorbance of the aqueous standards. For nitrate (NO_3_^−^) quantification, the PPAT supernatant was passed through a C18 column. Standards of KNO_3_ (potassium nitrate, 0.2–200 μM, Sigma) were prepared. The samples and standards (100 µL) were incubated with NADPH (Nicotinamide adenine dinucleotide phosphate; 100 µM), FAD (flavin adenine dinucleotide 5 µM), and NO_3_^−^ reductase (200 mUI/ml) for 1 h at 37°C. After this incubation period, the samples and standards were incubated with LDH (Lactate dehydrogenase 13.5 U/ml) and pyruvate (9 mM) for 30 min at 37°C. The Griess solution was added, and the absorbance was measured at 540 nm. The total nitrate plus nitrite concentrations were calculated from the calibration curve obtained through linear regression. The nitrate concentration in the PPAT supernatant was determined by subtracting the nitrite values.

### 2.9 Adenosine quantification

#### 2.9.1 Samples preparation

The PPAT was incubated with KHS (40 mg) for 30 min at 37°C without the prostate while being continuously bubbled with carboxygen (95% O_2_ and 5% CO_2_) to maintain a pH of 7.4. After this incubation period, the samples were collected and stored at -20°C until adenosine quantification. Trichloroacetic acid solution (5% TCA, 1:1) was added to the samples and the standard curve (1, 2.5, 5, 10, and 15 μg/L of adenosine). The samples were then centrifuged at 10,000 rpm for 10 min, and the supernatant was collected and transferred to a glass vial.

#### 2.9.2 Quantification

For chromatographic analysis, an Agilent Zorbax SB-AQ column with dimensions of 4.6 mm × 150 mm inner diameter and a particle diameter of 3.5 μm was utilized. The chromatographic separation was performed using the isocratic elution mode, with the mobile phase consisting of 0.1% (v/v) formic acid in water. The HPLC parameters employed were as follows: column temperature was set at 25°C, injection volume of 10 μL, mobile phase flow rate of 1 mL/min, and each sample/standard was analyzed for 20 min. A mass spectrometer, specifically the Micromass Quattro Micro API from Waters, equipped with a triple quadrupole (QqQ) analyzer and an electrospray ionization source, was utilized. Data acquisition was performed using the Masslynx 4.1 software. The sequential mass spectrometer was operated in the Multiple Reaction Monitoring (MRM) mode. The detection parameters in MRM mode for the analyte were as follows: precursor ion (268.16 m/z); product ion (118; 7–135.9 m/z); cone voltage (25 V); and collision energy (28–20 eV).

### 2.10 Functional assays

Initially, the ability of PPAT supernatant from obese mice to affect the reactivity of prostatic smooth muscle was assessed. For this, PPAT supernatant or KHS (vehicle) was incubated with prostatic strips from control and obese mice for 30 min after which concentration-response curves (CRC) to Phe (0.001–100 μM) or electrical field stimulation were obtained.

In a second series of experiments, 1 μM L-NAME, 10 µM 1400W, 10 µM ODQ, or 1 µM ZM241385 were incubated with PPAT supernatant or KHS for 30 min, after which the supernatant (1 ml) was added to strips of the prostatic tissue from obese mice. CRC to Phe was obtained as described above (Figure 1, protocol B). In additional experiments, the lability of the anticontractile activity of the PPAT supernatant was assessed by screening for activity after storing the supernatant at 4°C for 24, 48, and 72 h. The residual activity was assayed in the prostatic tissue collected from obese mice on the day of the experiment (Figure 1, protocol C).

Non-linear regression analysis was done using Prism v.8 (GraphPad Inc., San Diego, CA, United States) with the constraint that F = 0. All concentration-response data were fit to a logistic function of the form:

E = E_max_/([1 + (10^c^/10^x^)^n^] + F), where E is the response above basal, E_max_ is the maximum response produced by a given agonist, c is the logarithm of the pEC_50_ (the concentration of drug that produces a half-maximal response), x is the logarithm of the drug concentration, n is an exponential curve-fitting parameter that defines the slope of the CRC, and F is the response observed in the absence of added drug. The maximal responses (E_max_) were expressed as mN per milligram of tissue (mN/mg).

### 2.11 IL-6 and TNF-α quantification in PPAT supernatant

PPAT obtained from lean and obese mice was incubated with 1 ml of KHS and incubated for 30 min at 37°C, after which the supernatant was collected and assayed for IL-6 and TNF-a using commercial ELISA kits (R&D Systems, Minneapolis, MN, United States).

### 2.12 Human prostatic cell lines

The human normal prostatic epithelial cell line (PNT1-A) was kindly donated by Prof. Carmem Veríssima (Institute of Biology, UNICAMP). The cells were cultured in RPMI 1640 medium (product no. R0009, Vitrocell, Campinas, SP, Brazil), supplemented with 10% fetal bovine serum (product no. 12657–029, Gibco, Paisley, Scotland), and 1% antibiotic (penicillin-streptomycin, product no. 15140–122, Gibco). The cells were incubated in a humidified 5% CO_2_ atmosphere at 37°C and were used between passages 16–22. The human benign prostatic hyperplasia cell line (BPH-1) was purchased from Merck (Darmstadt, Hesse, Germany) and was cultured as described for PNT1-A. The cells were used between passages 9–11.

### 2.13 MTT assay

PNT1-A cells were cultured to confluency (24 h) in 96-well plates and then incubated with culture medium alone (control) or with PPAT supernatant from lean and obese mice. After incubation with or without PPAT supernatant for 24, 48, or 72 h, the cell medium was replaced with a solution of MTT (0.5 mg/mL) prepared in RPMI 1640 medium and incubated at 37°C for 4 h. After this period, the cells were washed in phosphate-buffered saline followed by the addition of DMSO for 1 h for the complete dissolution of the formazan crystals. The final absorbance was read at 560 nm using a microplate reader (Biotek Instruments, Winooski, VT, United States). The assay was conducted on three separate occasions, and for each instance, the PPAT obtained from two animals was utilized. Hence, a total of six animals were used in the entire experiment. The data were represented as percentages relative to the control (vehicle, KHS), which was considered 100%.

### 2.14 Cell proliferation

Cells (PNT1-A and BPH-1) were cultured to confluency (24 h) in 96-well plates and then incubated with culture medium alone (control) or with PPAT supernatant from obese mice. The cells were incubated with PPAT supernatant alone or with supernatant in the presence of 10 µM 1400W, 10 µM ODQ, 10 µM SQ22536, 1 µM ZM241385, or 1 µM MRS1754 and 1 μM L-NAME for 24, 48, or 72 h, after which cell proliferation was assessed using a Cyquant™ NF kit according to the manufacturer’s instructions (Invitrogen). The assay was conducted on three to four separate occasions, and for each instance, the PPAT obtained from two animals was utilized. Hence, a total of six to eight animals were used in the entire experiment. The data were represented as percentages relative to the control (vehicle, KHS), which was considered 100%.

### 2.15 Western blotting

PNT1-A cells were cultured in 12-well plates for 24 h followed by a further 72 h incubation with or without PPAT supernatant from obese mice. Staurosporine (100 mM) was used as a positive control. At the end of this incubation, the culture plates were centrifuged (20 min, 1500 *g*, and 4°C) and the supernatant was collected, and the protein concentrations were determined using the Bradford dye-binding assay. The samples were subsequently mixed with Laemmli buffer, heated at 100°C for 5 min, and then applied to 10% polyacrylamide gels for electrophoresis (SDS-PAGE). After electrophoresis, the proteins were transferred to nitrocellulose membranes (BioRad, San Diego, CA, United States), blocked with 3% BSA, and incubated overnight with specific primary antibodies for caspase-3 and cleaved caspase-3 (both diluted 1:1000; Cell signaling, Danvers, MA, United States) and β-actin (1:25.000; Sigma). Specific secondary peroxidase-conjugated antibodies (Santa Cruz Biotechnology, Dallas, TX, United States) and ECL-solution (BioRad) were used to detect the corresponding protein bands. Densitometry was done using a ChemicDoc^®^ MP Image System (BioRad). The level of cleaved caspase-3 protein was expressed in relation to that of caspase-3.

### 2.16 Statistical analysis

All numerical data were presented as the Mean ± Standard deviation (SD) (Michel et al., 2020). The “N” symbol indicates the number of animals used in the assays. Before starting the experimental protocols, we employed a specific formula to determine the appropriate group size to initiate our experiments. Considering our previous experience and literature research, we considered a standard deviation (SD) of 15% and aimed to detect a significant difference of 20% between groups. To ensure robust statistical analysis, we used a 95% confidence interval and a statistical significance level of 0.05, which are widely recommended for biological experiments. Based on the above information, the sample size was in the range of N = five to eight animals.

The student’s unpaired *t*-test was performed to compare animal, prostate, and PPAT weight, as well as glucose and insulin tolerance. For concentration and frequency response curves, the Shapiro-Wilk test was first used to assess the normality of data distribution. The comparisons between two groups were done using Student’s unpaired *t*-test, whereas comparisons among ≥3 groups were done using one-way analysis of variance (ANOVA), followed by the Bonferroni multiple comparisons test in Emax and EC_50_ values.

When using cell lineage protocols, the experiments were conducted on three to four separate occasions, utilizing the PPAT from two obese animals each time. Therefore, a total of N = six to eight animals were included in this assay. Consequently, the presented data represents the Mean ± SD of these independent instances. The student’s unpaired *t*-test was applied to western blotting analysis while the two-way ANOVA was used to assess cell proliferation and viability.

After completing each set of experimental protocols, we performed statistical analyses to assess the significance of the observed results. If a significant effect was detected, the corresponding protocols were terminated prematurely, as we had achieved our objective. Conversely, if indications suggested the presence of an expected effect, we continued the protocols to gather additional data. This approach and the ability to adjust group sizes align with the exploratory nature of the study while excluding hypothesis testing (Michel et al., 2020). It is important to note that no data were excluded from our analyses. All collected data points were taken into account, ensuring transparency and integrity throughout the study. The intended calculation of descriptive *p*-values (<0.05) necessitated a pre-planned minimum of five experiments per group, both in terms of the number of experiments and group sizes. The increasing number of animals was performed if these initial results did not reveal *p* < 0.05 but suggested the presence of an expected effect. If *p* values presented higher than 0.05 are not indicated. All data analyses were done using Prism v.8 (GraphPad).

## 3 Results

### 3.1 Morphometric and metabolic parameters

**FIGURE 2:**
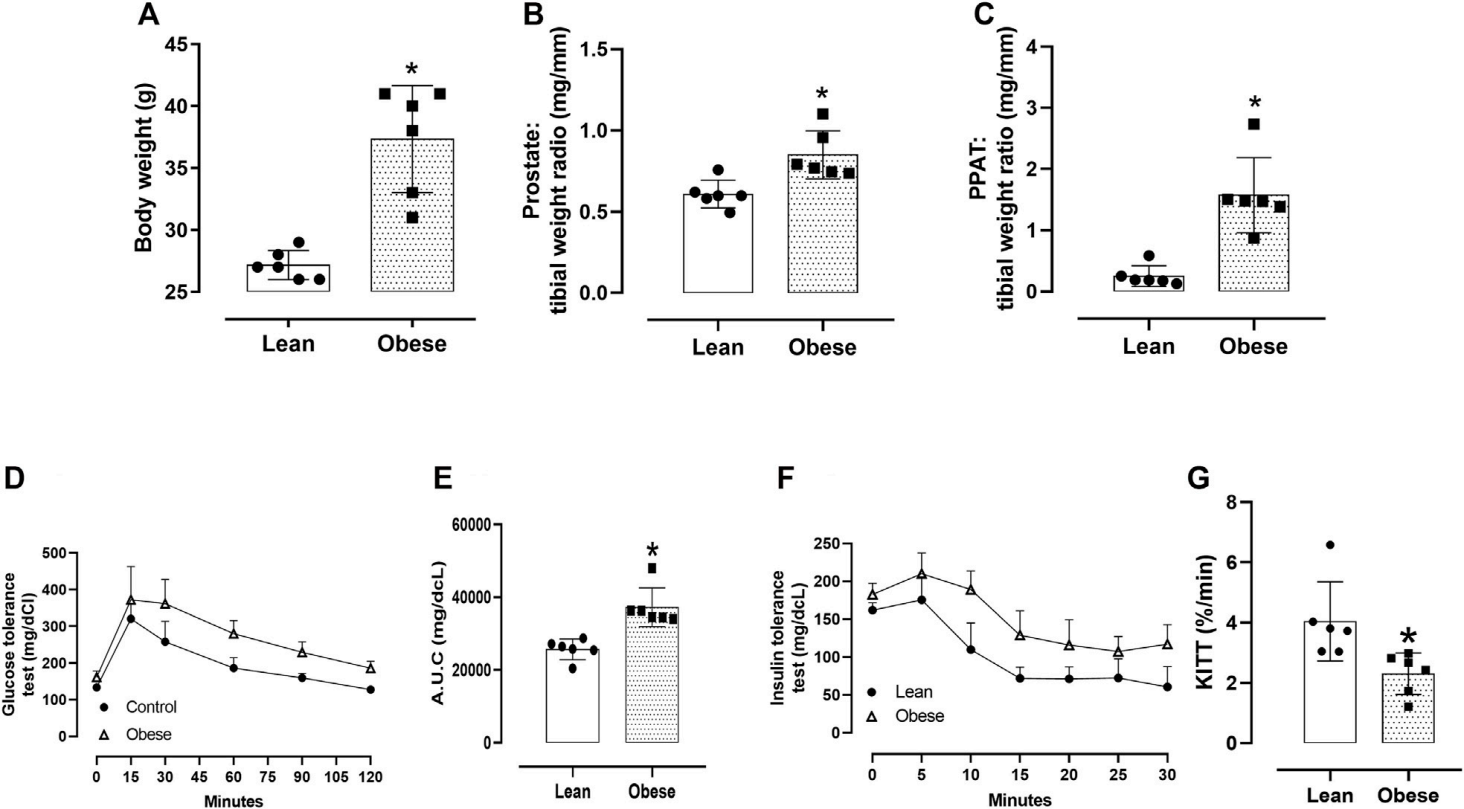
General characteristics. **(A)** Total body weight, **(B)** prostate weight, and **(C)** periprostatic adipose tissue (PPAT) weight. The prostate and PPAT weights were normalized relative to the tibial length of the mice. The columns are the mean ± SD of six mice/group. *p < 0.05, compared to lean mice. **(D)** Glucose tolerance test (GTT), with glucose concentrations expressed in mg/dL. Blood samples were obtained from lean and obese mice at 0, 15, 30, 60, 90, and 120 min. **(E)** The area under the glucose curve (A.U.C.) derived from the GTT test for lean and obese mice. **(F)** Insulin tolerance test (ITT), with glucose concentrations expressed in mg/dL. Blood samples were obtained from lean and obese mice at 0, 5, 10, 15, 20, 25, and 30 min. **(G)** Glucose decay constant (κITT; %/min). In panels **(E**–**G)**, the points and columns represent the mean ± SD (n = 5 mice/group). *p < 0.05 compared to lean mice in all cases (Student’s unpaired *t*-test).

Obese mice had a greater total body weight (*p* = 0.0001; Figure 2A; n = 6), prostate weight (*p* = 0.003; Figure 2B; n = 6), and PPAT relative weight (*p* = 0.0002; Figure 2C; n = 6) compared with the lean group. The tibial length, used for normalization, did not differ between the groups (18.2 ± 0.63 and 17.7 ± 0.08 mm, for lean and obese mice, n = 6/group, respectively). Obese mice were glucose intolerant, as shown by the greater area under the curve (AUC) in the GTT test (*p* = 0.01; Figures 2D,E; n = 6), and insulin intolerant, as shown by the higher circulating glucose concentrations and lower κITT (*p* = 0.007; Figures 2F,G; n = 6).

IL-6 and TNF-α were quantified in PPAT supernatant from lean and obese mice. The concentrations of TNF- α (525.5 ± 264.5 pg/ml) and IL-6 (313.5 ± 145.9 pg/mL) were significantly higher (*p* = 0.006 and *p* = 0.0001, respectively) in PPAT supernatant from obese mice than in PPAT supernatant from lean mice (39.8 ± 23.8 and 3.6 ± 2.4 pg/mL, respectively).

### 3.2 Prostate and PPAT histology

**FIGURE 3:**
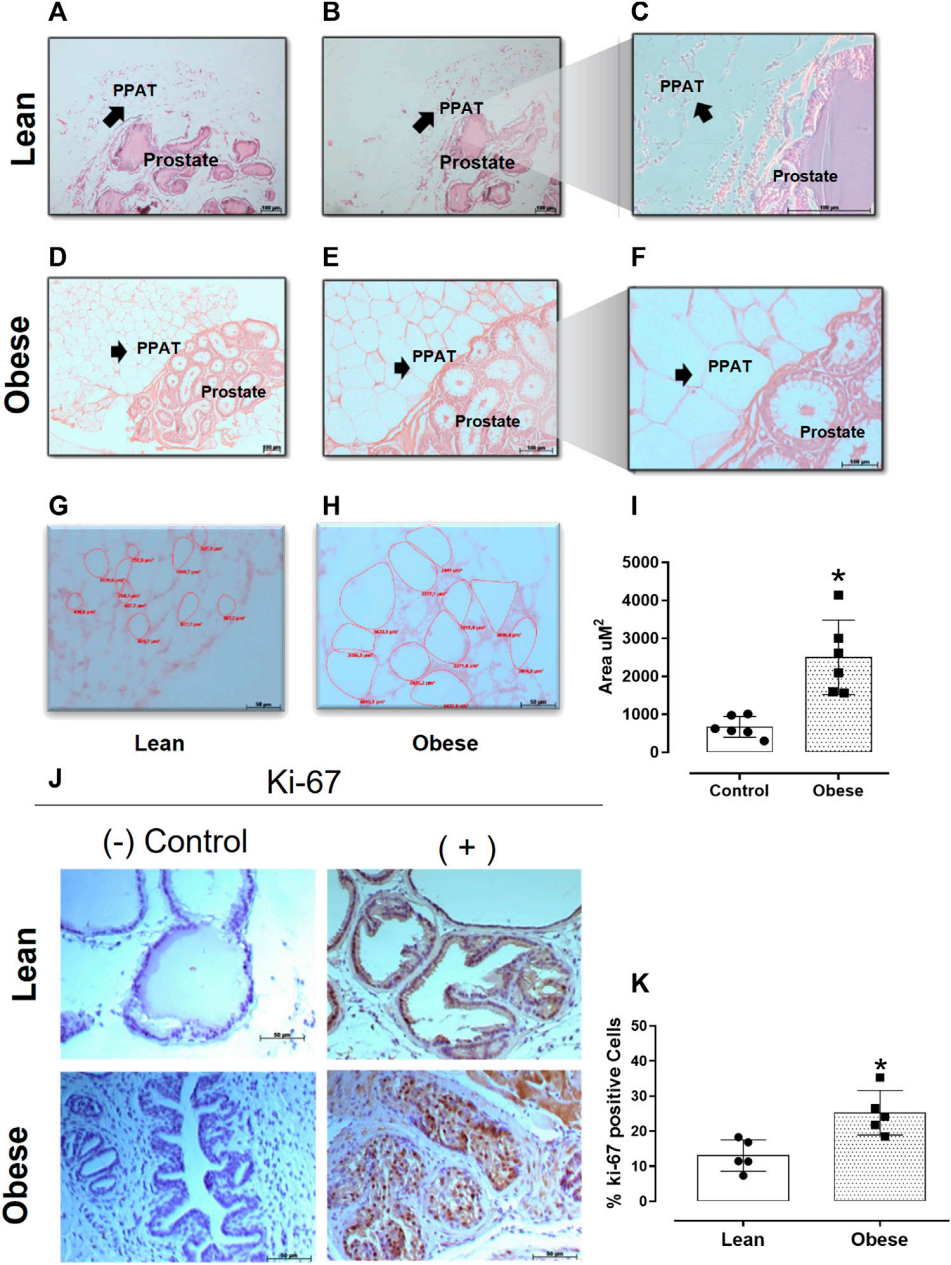
Hematoxylin-eosin-stained sections of prostate and periprostatic adipose tissue (PPAT) at different magnifications. 10x **(A,D)**, 20x **(B,E)**, and 40x **(C,F,G, and H)**. Scale bars in micrometers. **(I)** Adipocyte area. The area of 10 adipocytes of PPAT per slide was calculated and the results were expressed as the mean ± SD (n = 5). *p < 0.0007 compared to adipocytes from lean mice. **(J)** The representative photomicrographs of prostate tissue stained for the cell proliferation marker Ki-67. **(K)** Quantitative analysis of proliferating prostatic cells in lean and obese mice. The columns represent the mean ± SD (n = 5). *p < 0.05 compared to lean mice (Student’s unpaired *t*-test).

Prostate of lean mice showed a morphological structure characterized by a simple columnar epithelium with flat luminal borders and a thin layer of smooth muscle cells surrounding each acinus (Figures 3A–C). In contrast, the prostate of obese mice showed signs of epithelial hyperplasia (Figures 3D–F). Additionally, PPAT from obese mice (Figures 3G, H) showed an increase (∼60%) in the adipocyte area when compared to PPAT from lean mice (*p* = 0.0007, n = 6/group; Figure 3I).

### 3.3 Prostate cell proliferation

The proliferative index (based on Ki-67 expression) was significantly greater (by 93%; *p* = 0.004) in the prostate from obese mice (25.2 ± 2.8 cells, n = 5) when compared with prostate from lean mice (13 ± 3.5 cells, n = 5) (Figures 3J, K).

### 3.4 NOx^−^ quantification

**FIGURE 4:**
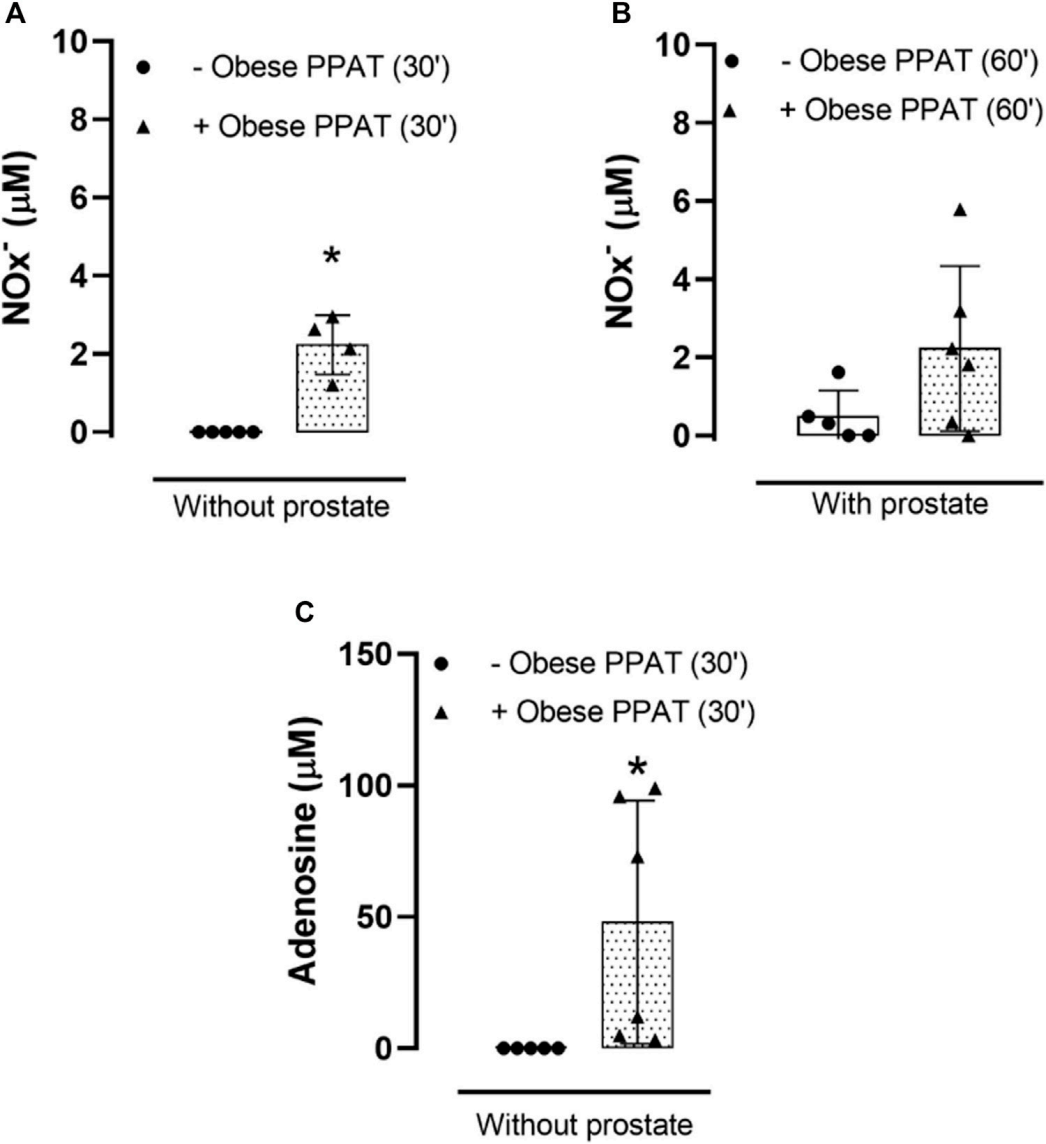
Total nitrite and nitrate (NOx-) and adenosine quantification. NOx^−^ were quantified in PPAT supernatant from obese mice or in the vehicle (KHS) for 30 min **(A)**, without prostate) and 60 min **(B)**, with prostate) at 37°C and continuously bubbled with carboxygen. **(C)** Adenosine was quantified in PPAT supernatant from obese mice (without prostate, 30 min) or in the vehicle (KHS) at 37°C and continuously bubbled with carboxygen. *p < 0.05 for–obese PPAT vs. + obese PPAT. The columns represent the mean ± SD (n = 5–6) (Student’s unpaired *t*-test).

The levels of NOx were significantly higher (*p* = 0.0001) in the PPAT supernatant (30 min, without prostate) when compared to the vehicle (Figure 4A). A non-significant (*p* = 0.056) trend toward increase was observed in the NOx levels in prostates incubated with PPAT supernatant (60 min) in comparison with prostates incubated with the vehicle (60 min, KHS) (Figure 4B).

### 3.5 Adenosine quantification

The levels of adenosine were significantly higher (*p* = 0.02) in the PPAT supernatant (30 min, without prostate) when compared to the vehicle, KHS (Figure 4C).

### 3.6 PPAT from obese mice releases anticontractile substances

**FIGURE 5:**
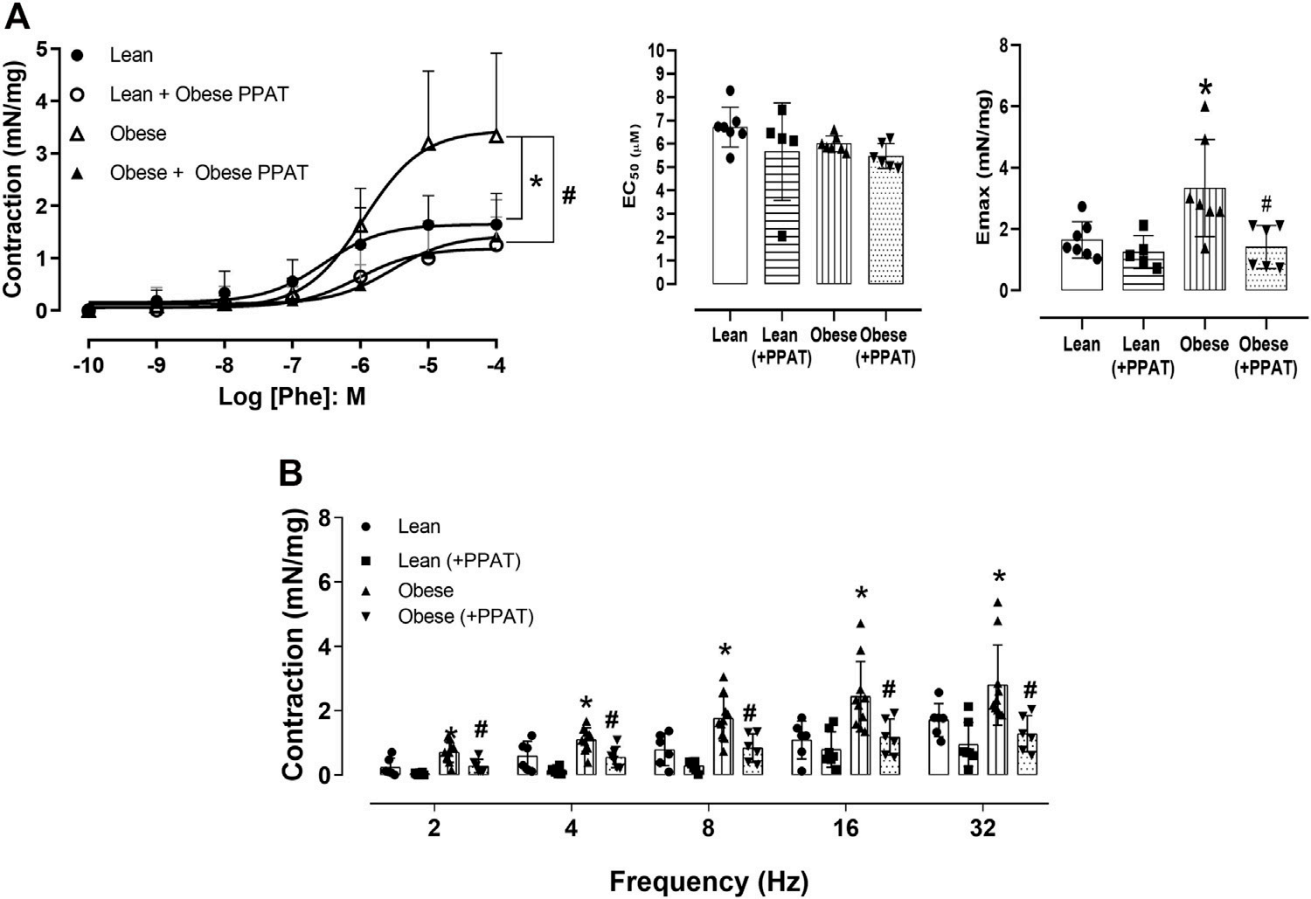
Functional reactivity assays. **(A)** Concentration-response curves to phenylephrine (Phe, n = 7) or **(B)** Electrical field stimulation of the prostatic tissue from lean (n = 6) and obese (n = 10) mice in the absence (KHS alone) and the presence of supernatant from periprostatic adipose tissue (+PPAT) of obese mice. PPAT from obese mice was incubated with KHS for 30 min at 37°C with carboxygen, after which the supernatant was collected and tested for biological activity. The points and columns represent the mean ± SD of the indicated number of mice. *p < 0.05 for control vs. obese and ^#^ *p* < 0.05 for obese vs. obese + PPAT (Student’s unpaired *t*-test, or one-way ANOVA followed by the Bonferroni multiple comparisons test.) The experiment with PPAT from lean animals was not tested for ethical and cost reasons involved.

The cumulative addition of Phe (Figure 5A) produced concentration-dependent contractions in prostatic tissue from both groups although the magnitude of the contractions was significantly greater (*p* = 0.01) in the prostate from obese mice (3.33 ± 1.58 mN/mg, N = 7) compared to lean mice (1.64 ± 0.59 mN/mg, n = 7). The incubation of PPAT supernatant with prostatic strips from lean mice caused a 2.8-fold rightward shift although this did not reach statistical significance (pEC_50_: 6.43 ± 0.30 vs. 5.98 ± 0.33 and E_max_: 1.64 ± 0.59 and 1.25 ± 0.53 mN/mg, in the absence and presence of PPAT, respectively, n = five to seven, *p* > 0.05) (Figure 5A). In contrast, the incubation of PPAT supernatant with prostatic strips from obese mice reduced Phe-induced contractions by ∼58% (from 3.33 ± 1.58 mN/mg to 1.41 ± 0.70 mN/mg, n = 6, *p* = 0.006, Figure 4A). Electrical stimulation-induced neurogenic contraction in prostatic tissue from both groups of mice. The amplitude of contraction was significantly (*p* < 0.05) greater in the prostate of obese (N = 7) compared to lean mice (N = 6) at 2–32 Hz (Figure 5B). Incubation with PPAT supernatant significantly reduced the EFS-induced contraction in the prostate of obese mice for all frequencies (*p* < 0.05, N = 6, Figure 5B). In the prostate of lean mice, the PPAT supernatant from obese mice also reduced the EFS-induced contractions, but the reductions were not significant (*p* > 0.05).

### 3.7 The anticontractile activity of supernatant from PPAT of obese mice involves the release of NO and adenosine

The cyclic nucleotides cAMP and cGMP are the main second messengers that promote the relaxation of prostatic smooth muscle. To investigate this possibility, the PPAT supernatant from obese mice was incubated for 30 min both with and without L-NAME, 1400W, ODQ, or ZM241385, and then added to the prostate of obese mice. Preliminary experiments showed that at the concentrations tested, these antagonists and inhibitors alone did not significantly interfere with the potency or maximal responses to Phe in prostatic tissue from obese mice (Table 1).

**TABLE 1:** Potency (pEC_50_) and maximal response (E_max_) values of phenylephrine in isolated prostate from lean or obese mice in the absence (-) and presence (+) of L-NAME, 1400W, ODQ, and ZM 241385.

		pEC_50_	E_max_ (mN/mg)	N
**Lean**	-L-NAME (1 μM)	6.77 ± 0.8	1.71 ± 0.57	5
	+L-NAME (1 μM)	6.70 ± 0.8	1.76 ± 0.72	5
	-1400W (10 μM)	6.52 ± 0.4	2.30 ± 0.32	5
	+1400W (10 μM)	6.57 ± 1.2	1.97 ± 1.1	5
	- ODQ (10 μM)	6.63 ± 0.7	1.97 ± 0.75	5
	+ ODQ (10 μM)	6.13 ± 0.4	2.25 ± 0.81	5
	-ZM 241385 (1 μM)	6.41 ± 0.2	2.02 ± 0.38	5
	+ZM 241385 (1 μM)	6.90 ± 0.9	2.02 ± 0.81	5

**Obese**	-L-NAME (1 μM)	5.95 ± 0.5	3.33 ± 1.58	7
	+L-NAME (1 μM)	6.22 ± 0.3	2.68 ± 0.44	6
	-1400W (10 μM)	5.95 ± 0.5	3.33 ± 1.58	7
	+1400W (10 μM)	5.86 ± 0.41	2.77 ± 0.10	6
	- ODQ (10 μM)	6.39 ± 0.7	3.40 ± 1.31	6
	+ ODQ (10 μM)	6.84 ± 0.53	2.61 ± 0.65	6
	-ZM 241385 (1 μM)	5.95 ± 0.5	3.33 ± 1.58	7
	+ZM 241385 (1 μM)	6.71 ± 0.9	2.90 ± 0.78	7

**FIGURE 6:**
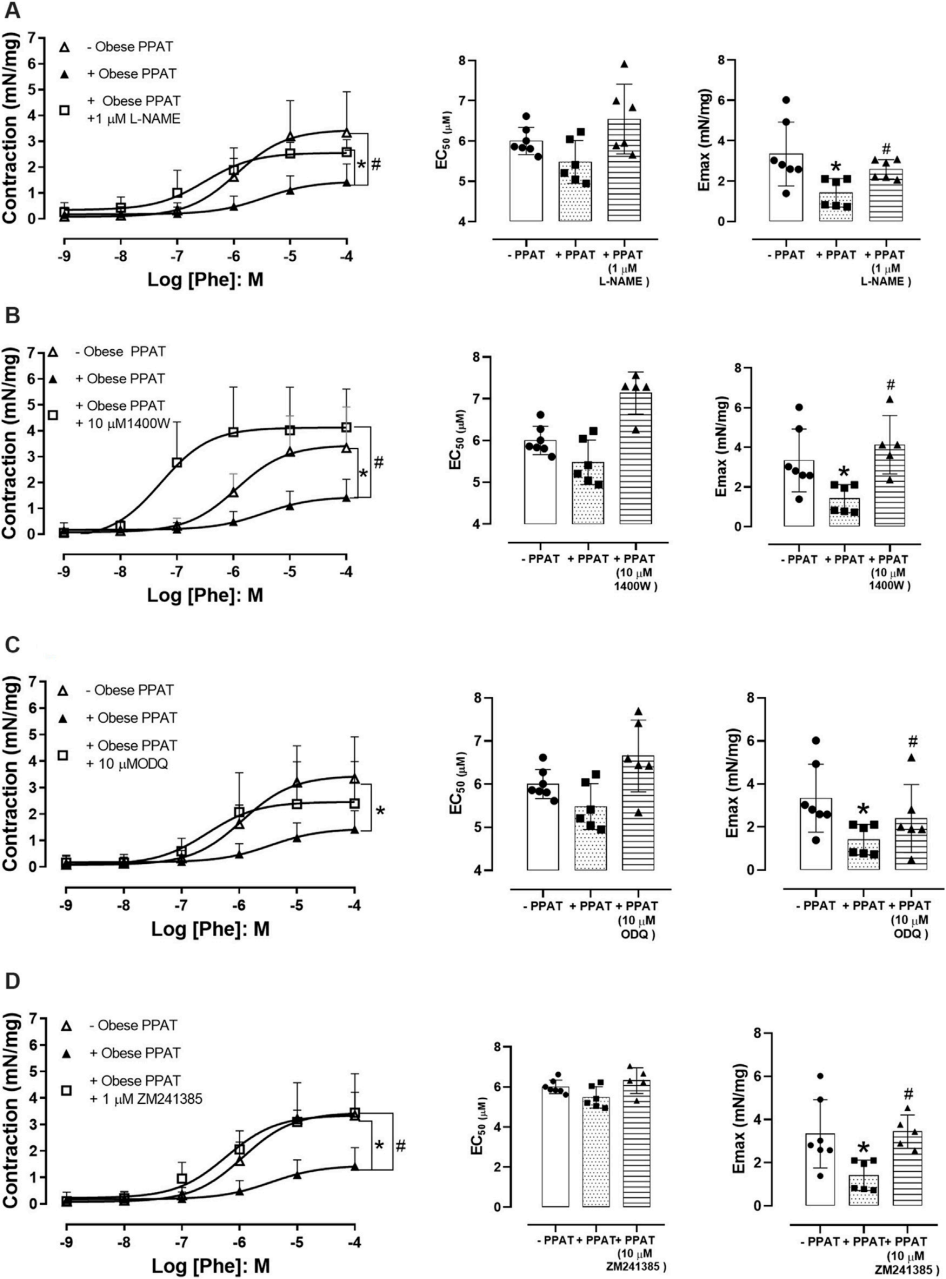
Functional reactivity assays in the presence of inhibitors/antagonists. Concentration-response curves to phenylephrine (Phe) in prostatic tissue from obese mice incubated in the absence or presence of periprostatic adipose tissue (PPAT) alone (+PPAT), and with **(A)** L-NAME, a non-selective NOS inhibitor (n = 6), **(B)** 1400W, an inhibitor of inducible NOS (n = 5), **(C)** ODQ, a soluble guanylate cyclase inhibitor (n = 6), and **(D)** ZM241385, an adenosine A_2A_ receptor antagonist (n = 5). The points represent the mean ± SD of the indicated number of mice. *p < 0.05 for–PPAT vs. + PPAT and ^#^ *p* < 0.05 for + PPAT vs. + PPAT with inhibitors (one-way ANOVA followed by the Bonferroni multiple comparisons test).

Figure 6 shows that the co-incubation of PPAT with L-NAME (N = 6), 1400W (N = 5), ODQ (N = 6), and ZM241385 (N = 5) followed by testing the resulting supernatant reversed or prevented the anticontractile activity of PPAT supernatant to varying degrees (panels A to D, respectively). This finding suggested that NO and adenosine release or formation are involved in the anticontractile response. Since PPAT supernatant from obese mice did not significantly attenuate Phe-induced contractions in prostatic tissue from lean mice, these inhibitors were tested only in the prostate of obese mice.

### 3.8 PPAT supernatant from obese mice increases the viability of PNT1-A cells

**FIGURE 7:**
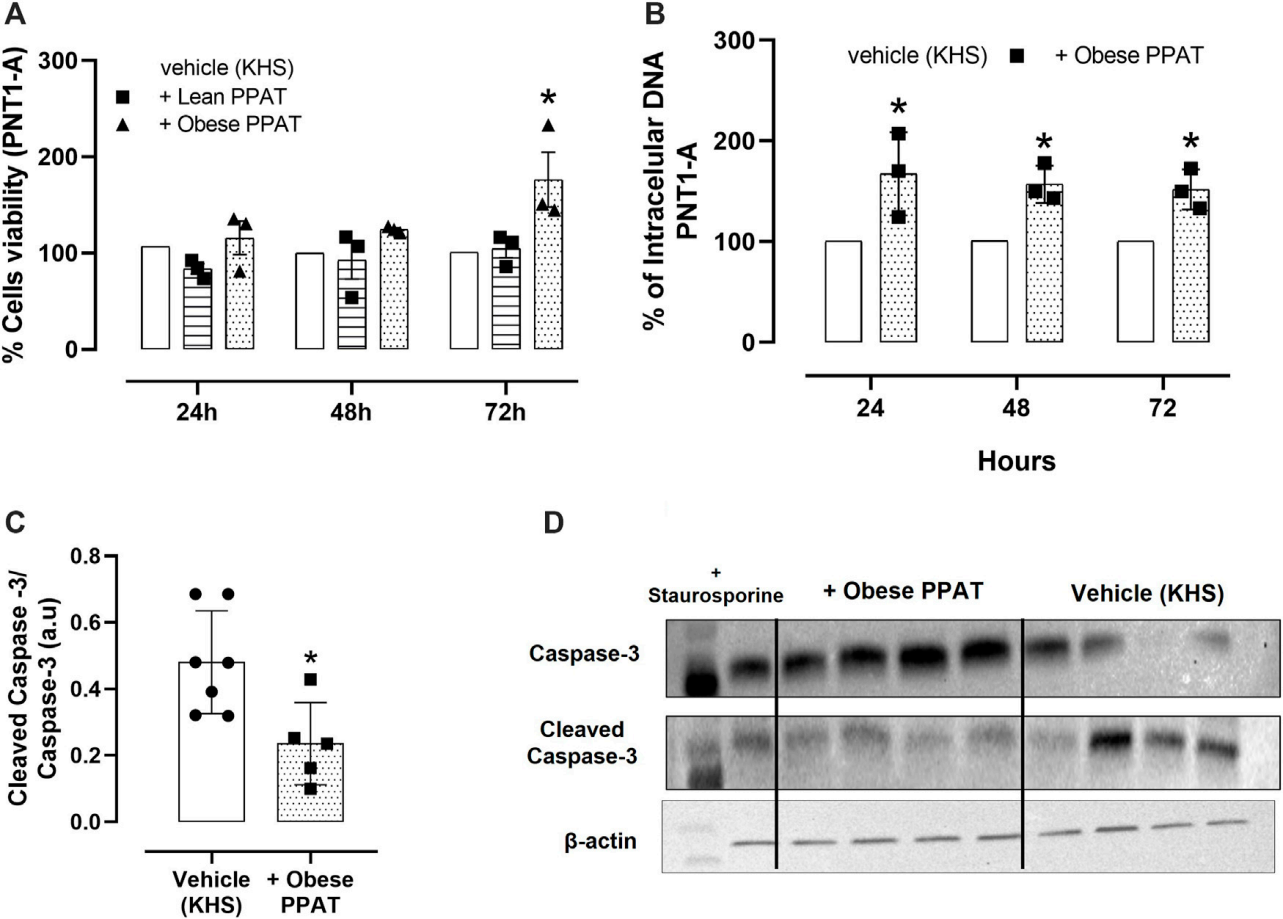
Cell-based assays with normal epithelial cells. Normal human epithelial cells (PNT1-A) viability (% of the vehicle) in the presence of lean periprostatic adipose tissue (+ Lean PPAT) and obese periprostatic adipose tissue (+Obese). **(A)** Cell proliferation in PNT1-A in the absence (vehicle) and presence of supernatant from periprostatic adipose tissue (PPAT) of obese mice. The cells were incubated with and without PPAT for 24 h, 48 h, and 72 h, after which cell proliferation was assessed using CyQUANT^®^ assay kits. **(B)** The columns represent the mean ± SD (Student´s unpaired *t*-test or one-way ANOVA followed by the Bonferroni multiple comparisons test) of the three separate occasions. Each time, the PPAT was obtained from two animals. Therefore, in panels **(A)** and **(B)**, a total of N = 6 animals were included in this assay. The effect of PPAT supernatant on the level of cleaved caspase-3 protein in PNT1-A cells. The level of cleaved caspase-3 was normalized relative to caspase-3 protein expression levels, and the values are expressed in arbitrary units (a.u.). **(C)** Representative images of the WB analysis showing + obese PPAT and Vehicle. **(D)** The columns represent the mean ± SD, N = 6 animals (Student´s unpaired *t*-test or one-way ANOVA followed by the Bonferroni multiple comparisons test).

The supernatant of PPAT from obese mice enhanced PNT1-A cell viability after incubation for 72 h when compared to cells incubated only with the vehicle alone. The supernatant of PPAT from lean mice had no effect on cell viability compared to cells incubated with the vehicle alone (Figure 7A). Based on these findings, the experiments involving cell proliferation were done only with a supernatant of PPAT from obese mice.

### 3.9 PPAT supernatant stimulates the proliferation of PNT1-A cells and decreases the level of cleaved caspase-3 protein

The incubation of PNT1-A cells (normal human epithelial cells) with PPAT supernatant from obese mice for 24, 48, and 72 h significantly increased (*p* = 0.006; *p* = 0.02; *p* = 0.04, respectively) the cell proliferation at all time intervals (increases of 67.4%, 57%, and 51.8% after 24 h, 48 h, and 72 h, respectively) when compared to cells incubated with vehicle solution (KHS, Figure 7B). In addition, incubation with PPAT supernatant for 72 h significantly reduced the level of cleaved caspase-3 protein when compared to cells treated with vehicle solution alone (Figure 7C).

### 3.10 PPAT supernatant stimulates the proliferation of BPH-1 cells

**FIGURE 8:**
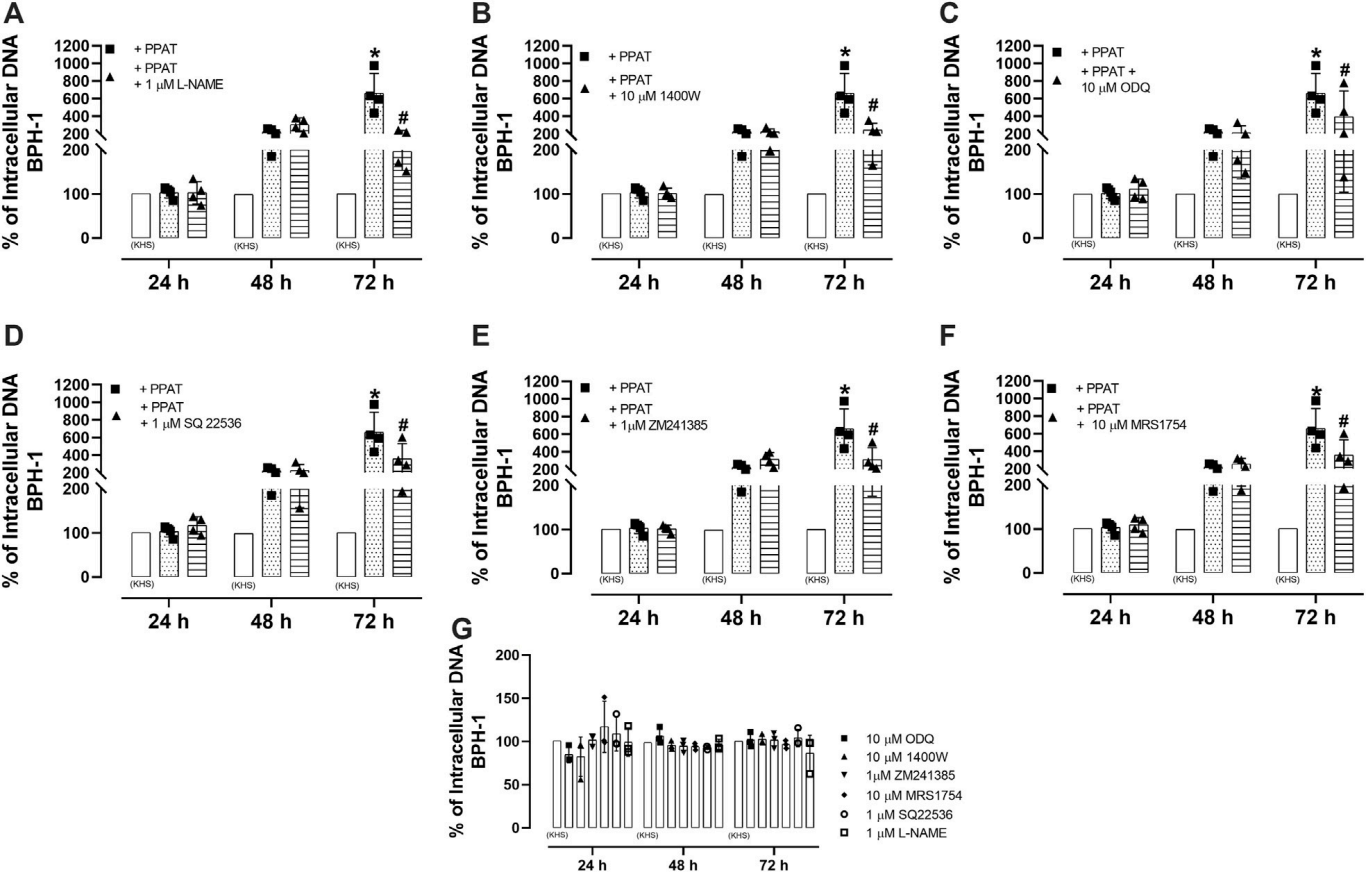
Cell-based assays with hyperplastic epithelial cells. Cell proliferation in a human benign prostatic hyperplasia cell line (BPH-1) in the absence (vehicle KHS) and presence of supernatant from periprostatic adipose tissue (PPAT) of obese mice (+PPAT) incubated alone or with **(A)** L-NAME, a non-selective NOS inhibitor, **(B)** 1400W, an inhibitor of inducible NOS, **(C)** ODQ, a soluble guanylate cyclase inhibitor, **(D)** SQ 22,536, an inhibitor of adenylate cyclase, **(E)** ZM241385, an adenosine A_2A_ receptor antagonist, and **(F)** MRS 1754, an adenosine A_2B_ receptor antagonist. **(G)** The proliferation of BPH-1 cells in the presence of inhibitors or antagonists alone. None of the compounds had any effect on cell proliferation at the concentrations tested. In all cases, cell proliferation was assessed using CyQUANT^®^ assay kits. The columns represent the mean ± SD of three to four occasions (each done in triplicate). Each time, the PPAT was obtained from two animals. Therefore, a total of N = six to eight animals were included in this assay. *p < 0.05 for the vehicle vs. + PPAT and ^#^ *p* < 0.05 for + PPAT vs. + PPAT plus inhibitors (two-way ANOVA followed by the Bonferroni multiple comparisons test).

Figure 7A shows that the incubation of BPH-1 cells (hyperplasic human epithelial cells) with PPAT supernatant from obese mice for 24, 48, and 72 h resulted in significant (*p* < 0.0001) cell proliferation after 72 h compared to cells incubated with the vehicle alone. The incubation of PPAT with L-NAME, 1400W, ODQ, SQ22,536, ZM241385, and MRS1754 (Figures 8A–F) significantly attenuated the cell proliferation seen after 72 h. None of these antagonists or inhibitors affected cell proliferation when tested alone (Figure 8G).

## 4 Discussion

Our results showed that the supernatant of PPAT from obese mice reduced the contractile response induced by Phe and favored the human epithelial cell proliferation in part due to the release of NO and adenosine.

Adipose tissue is classified as white (WAT) and brown (BAT) or beige based on the morphology and function of these lipid deposits. WAT consists of non-thermogenic, energy-storing fat cells composed of a unilocular lipid droplet that occupies most of the adipocyte area. On the other hand, BAT is thermogenic and formed by multilocular lipid droplets dispersed throughout a cytoplasm enriched with mitochondria (Lu et al., 2010; Jeremic et al., 2017). PPAT from obese mice shares similarities with WAT in that it consists of a unilocular lipid droplet. Obesity increases the weight of PPAT and the adipocyte area. PPAT from obese mice has an inflammatory profile similar to that of WAT, which has higher levels of TNF-a and IL-6 when compared to PPAT from lean mice (Alexandre et al., 2018; Bhardwaj et al., 2019).

There is preclinical and clinical evidence that, besides aging, other modifiable factors such as diabetes, metabolic syndrome, dyslipidemia, and hypertension contribute to the development of BPH and LUTS (Mónica and Antunes, 2018; Passos et al., 2021). On the other hand, the correlation between LUTS/BPH and obesity remains controversial. While many studies reinforce the idea that obesity increases the risk of BPH and LUTS (Kim et al., 2021; Chen et al., 2022; Qasrawi et al., 2022; Wang et al., 2022), others have reported a similar pattern of voiding function in lean, overweight, and obese patients (Bauer et al., 2022). In a previous report (Calmasini et al., 2017), we showed that obesity caused prostate hyperplasia, insulin resistance, and greater contractile responses to a_1_-adrenoceptor agonists. Our findings described in this study confirm previous results regarding the enhanced contractile activity in response to Phe and transmural electrical stimulation in the prostate of obese mice.

Since PPAT is in direct contact with the prostate and prostatic urethra, our initial hypothesis was that PPAT from obese mice could release contractile substances capable of causing prostatic hypercontractility in tissue from obese mice. However, when the supernatant of PPAT from obese mice was tested *in vitro* there was a ∼58% reduction in the amplitude of contraction induced by Phe and transmural electrical stimulation.

Since cyclic AMP (cAMP) and cyclic GMP (cGMP) are the major second messengers that promote prostatic smooth muscle relaxation (Mónica and Antunes, 2018), we examined the potential involvement of these signaling pathways in the anticontractility activity of PPAT. To accomplish this, PPAT was co-incubated with inhibitors of NOS (L-NAME or 1400W), soluble guanylate cyclase (ODQ), and adenylate cyclase (SQ22,536), or with the adenosine receptor antagonist (ZM241385). The co-incubation of PPAT with these inhibitors markedly attenuated the inhibitory effect induced by PPAT, indicating a role for NO and adenosine. Another interesting point to be discussed is that the supernatant incubated with 1400W produced a marked potentiation in Phe-induced contraction. Although it is only a speculation, the levels and/or activity of iNOS may be higher in the different PPAT cell types (adipocyte, inflammatory, endothelial, vascular smooth muscle cells, among others) and, hence, the blockade of NO by 1400W promoted a pronounced potentiation. In the perivascular adipose tissue (PVAT) from animals that received a high-carbohydrate diet, the expression of iNOS, among other targets, was markedly increased (dos Reis Costa et al., 2021). Other studies showed that PVAT reduces the contractile response induced by the adrenergic agonists in part due to the release of NO (Victorio et al., 2016; Gonzaga et al., 2018). In the latter two studies, the incubation of NOS inhibitors with PVAT also resulted in leftward shifts in the contraction induced by phenylephrine.

The levels of total nitrite and nitrate were higher in the PPAT supernatant (before adding to the isolated prostate) both before and at the end of the concentration-response curve of phenylephrine, thus showing the presence of NO metabolites in the whole experimental protocol. As the half-life of NO is short, the source of NO comes from the cellular components of PPAT. Although we have not characterized the specific cell types present in the PPAT supernatant, previous studies have shown that in addition to adipocytes, preadipocytes, fibroblast, macrophages, lymphocytes, and endothelial cells are the main cellular components found in the adipose tissue (Laurent et al., 2016; Vijay et al., 2020). All these cells express NOS and produce NO (Mónica et al., 2016).

The inhibitory effect observed here is similar to that described in other adipose depots. For example, the Phe-induced contractions of aortic rings from Balb/c mice that received a high-carbohydrate diet for 4 weeks were significantly attenuated only in vessels isolated with PVAT. The anticontractile response induced by PVAT was absent when the aorta was incubated with an angiotensin-converting enzyme inhibitor, a Mas receptor antagonist, an angiotensin AT_2_ receptor antagonist, and NOS inhibitors, thus suggesting the role of angiotensin II (ANG-II, acting through AT_2_ receptors), ANG_1-7_ (acting through Mas receptors), and NO signaling pathways (Dos Reis Costa et al., 2021). In low-density lipoprotein receptor (LDLr)- knockout mice (KO), the absence of PVAT impaired the relaxation induced by acetylcholine and insulin (Baltieri et al., 2018). Based on these findings, the authors proposed a protective role for PVAT in the initial phase of obesity to curb the associated atherosclerosis and ischemia and to preserve vascular function.

The clinical features of BPH result from static and/or dynamic factors and there is no evidence of neoplastic involvement (Liu et al., 2020). Most of the studies that have assessed the role of PPAT in the prostate were done in prostate cancer cell lines or in primary cell cultures from patients diagnosed with prostate cancer or in tissues from patients with prostate cancer (Finley et al., 2009; Ribeiro et al., 2012; Nassar et al., 2018; Romingué et al., 2022). A recent study showed that human PPAT differs from other adipose depots. For example, vessel density and HIF-2a protein expression in human PPAT are lower and higher, respectively, than in the abdominopelvic adipose tissue, suggesting that PPAT can lead to hypoxia. In this study, the authors observed that PPAT did not expand in obesity, perhaps because of the higher content of the extracellular matrix. Proinflammatory cytokines and macrophages and lower adiponectin levels are among the major factors that promote prostate disorders (Rouminguié et al., 2022). It remains to be determined whether PPAT from rodents has similar characteristics to that of humans.

As shown in this study, PPAT from obese mice released NO and adenosine and had higher levels of IL-6 and TNF-a compared to PPAT from lean mice. The role of adipocytokines in the proliferation of normal and prostatic cancer cells has been extensively explored (Nassar et al., 2018; La Civita et al., 2021). Since mouse cytokines do not activate human receptors (Lokau and Garbers, 2020) and NO and adenosine are conserved among species and are released under stress (Fukumura et al., 2006), a second hypothesis of this work was that NO and/or adenosine could interfere with the prostate cell viability.

NO is produced by three NOS isoforms (eNOS, nNOS, and iNOS) and activates soluble guanylate cyclase to produce cGMP (Förstermann and Sessa, 2012). A dual role (proliferative and antiproliferative) for NO on cell viability has been demonstrated and depends on the concentration of NO released and the duration of exposure to this mediator (Fukumura et al., 2006). Acute exposure (6 h) to the long-acting NO donor (DETA/NO, 300–500 µM) exerted an antiproliferative effect in human prostate epithelial cells (RWPE-1) by arresting the cells in the G1 phase, whereas longer exposures to DETA/NO (300–500 µM) (24 h, for 4 weeks) caused RWPE-2 proliferation, migration, and invasion in serum-free medium. Similar findings were observed in prostate cancer cell lines (Sarma et al., 2007).

Adenosine, an adenine-based nucleoside produced by the breakdown of intra- and extracellular adenine-based nucleotides, acts through four receptor subtypes (A_1_, A_2A_, A_2B,_ and A_3_). The A_2_ receptors are linked to a stimulatory G-protein (Gs), the activation of which leads to increased intracellular production of cAMP (Borea et al., 2018). In the prostate cancer cell line PC-3, the non-selective (NECA) and selective (BAY60-6583) A_2B_ receptor agonist increased PC-3 proliferation that was attenuated by specific siRNA for this receptor and by the A_2B_ receptor antagonist PSB603 (Wei et al., 2013). As shown in this study, the supernatant of PPAT from obese mice enhanced and decreased the proliferation and apoptosis (lower levels of cleaved caspase-3/caspase 3) of normal (PNT1-A) and increased the proliferation of hyperplasic human epithelial prostatic (BPH-1) cell lines after 72-h of incubation period. The co-incubation of PPAT with NOS-cGMP signaling pathway inhibitors (L-NAME, 1400W or ODQ) and adenosine A_2A_ (ZM241385) or A_2B_ (MRS1754) receptor antagonists, significantly reduced the proliferation of these cells, indicating the participation of NO and adenosine in this phenomenon.

The present study has some limitations. First, it was not possible to include the PPAT supernatant from lean mice in the functional assays. Second, based on the results of prostate contractility, we cannot conclude that PPAT directly leads to LUTS/BPH. However, this does not rule out the possibility that PPAT may have a negative impact on prostate function. While PPAT showed an anticontractile effect on the isolated prostate, it simultaneously promoted cell proliferation and reduced apoptosis. Furthermore, higher levels of TNF-α and IL-6 were observed in the supernatant, indicating potential interference with other aspects of prostate function beyond smooth muscle contractility. It remains to be determined whether longer periods of obesity can alter the phenotype of PPAT, potentially transitioning from an anticontractile to a pro-contractile state.

## 5 Conclusion

In conclusion, our study showed that, *in vitro*, the release of NO and adenosine of PPAT from obese mice fed with a high-fat diet for 12 weeks produced an anticontractile response in isolated prostate from mice and favored the proliferation of the human epithelial cell line. These findings provide an opportunity to investigate whether the blockade of NO and adenosine released from PPAT improve prostate function *in vivo.*

## Data Availability

The original contributions presented in the study are included in the article/supplementary material, further inquiries can be directed to the corresponding author.
